# Acute hyperglycemia suppresses left ventricular diastolic function and inhibits autophagic flux in mice under prohypertrophic stimulation

**DOI:** 10.1186/s12933-016-0452-z

**Published:** 2016-09-22

**Authors:** Jiahe Xie, Kai Cui, Huixin Hao, Yingxue Zhang, Hairuo Lin, Zhenhuan Chen, Xiaobo Huang, Shiping Cao, Wangjun Liao, Jianping Bin, Masafumi Kitakaze, Yulin Liao

**Affiliations:** 1 State Key Laboratory of Organ Failure Research, Department of Cardiology, Nanfang Hospital, Southern Medical University, 1838 Guangzhou avenue north, Guangzhou, 510515 China; 2 Department of Oncology, Nanfang Hospital, Southern Medical University, Guangzhou, 510515 Guangdong China; 3 Cardiovascular Division of the Department of Medicine, National Cerebral and Cardiovascular Center, Osaka, Japan

**Keywords:** Acute hyperglycemia, Diastolic function, Myocardial hypertrophy, Autophagic flux

## Abstract

**Background:**

Left ventricular (LV) dysfunction is closely associated with LV hypertrophy or diabetes, as well as insufficient autophagic flux. Acute or chronic hyperglycemia is a prognostic factor for patients with myocardial infarction. However, the effect of acute hyperglycemia on LV dysfunction of the hypertrophic heart and the mechanisms involved are still unclear. This study aimed to confirm our hypothesis that either acute or chronic hyperglycemia suppresses LV diastolic function and autophagic flux.

**Methods:**

The transverse aortic constriction (TAC) model and streptozocin-induced type 1 diabetic mellitus mice were used. LV function was evaluated with a Millar catheter. Autophagic levels and autophagic flux in the whole heart and cultured neonatal rat cardiomyocytes in response to hyperglycemia were examined by using western blotting of LC3B-II and P62. We also examined the effect of an autophagic inhibitor on LC3B-II and P62 protein expression and LC3 puncta.

**Results:**

In mice with TAC, we detected diastolic dysfunction as early as 30 min after TAC. This dysfunction was indicated by a greater LV end-diastolic pressure and the exponential time constant of LV relaxation, as well as a smaller maximum descending rate of LV pressure in comparison with sham group. Similar results were also obtained in mice with TAC for 2 weeks, in addition to increased insulin resistance. Acute hyperglycemic stress suppressed diastolic function in mice with myocardial hypertrophy, as evaluated by invasive LV hemodynamic monitoring. Mice with chronic hyperglycemia induced by streptozocin showed myocardial fibrosis and diastolic dysfunction. In high glucose-treated cardiomyocytes and streptozocin-treated mice, peroxisome proliferator-activated receptor-γ coactivator 1α was downregulated, while P62 was upregulated. Autophagic flux was also significantly inhibited in response to high glucose exposure in angiotensin-II treated cardiomyocytes.

**Conclusions:**

Acute hyperglycemia suppresses diastolic function, damages mitochondrial energy signaling, and inhibits autophagic flux in prohypertrophic factor-stimulated cardiomyocytes.

## Background

Left ventricular (LV) diastolic dysfunction is present in various cardiovascular diseases such as hypertension [1], myocardial hypertrophy [2–4], coronary heart disease [5], and diabetes mellitus (DM) [6, 7]. Worsening of diastolic dysfunction is a predictor of mortality in cardiovascular diseases and some severe non-cardiovascular diseases, including septic shock and acute pancreatitis [8–10]. The detrimental role of chronic hyperglycemia and DM on diastolic function has been well recognized [11]. The prognostic value of acute hyperglycemia in patients with acute myocardial infarction has also attracted attention of clinical investigators [12]. However, whether acute hyperglycemia is a factor that worsens diastolic dysfunction is unclear, and if so, the underlying mechanisms are unknown. Addressing these issues is of clinical importance for avoiding deterioration of diastolic dysfunction in patients with severe diseases.

Glucose deprivation causes autophagy. Therefore, hyperglycemia is likely to inhibit autophagy [13]. Insufficient autophagy is thought to cause or promote heart failure [14–16]. However, a previous report showed that increased autophagy contributes to LV diastolic dysfunction in pulmonary arterial hypertension [17]. Glucose is the main source of energy in the failing heart. Peroxisome proliferator-activated receptor-γ coactivator (PGC)-1α, a transcriptional coactivator, is beneficial for heart failure by inducing a change in phenotype towards oxidative metabolism of energy [18]. The association between PGC-1α and autophagy in cardiomyocytes that are exposed to high glucose is unclear. However, several lines of evidence in non-cardiomyocytes have shown that PGC-1α promotes autophagy [19, 20]. Based on all of these findings, we hypothesize that acute hyperglycemic stress suppresses diastolic function in preexistent heart disease by downregulating PGC-1α and inhibiting autophagic flux.

In the present study, we investigated the effect of acute hyperglycemia on LV diastolic function in a pressure-overload model with preexistent diastolic dysfunction and insulin resistance. We also investigated the potential mechanisms related to PGC-1α and autophagic flux in cultured cardiomyocytes. Although echocardiographic evaluation of LV diastolic dysfunction is a routine procedure in clinical practice, its accuracy is inferior to cardiac catheterization. Therefore, we used invasive LV hemodynamic parameters to evaluate diastolic function in this study.

## Methods

### TAC model

C57BL/6 male mice (9–10 weeks old) were subjected to TAC or sham operation as previously described [2, 21]. Briefly, prior to surgery, mice were anesthetized with a combination of ketamine (100 mg/kg) and xylazine (5 mg/kg) via intraperitoneal injection (ip), after intubation and artificial respiration, the chest-open was then opened via the second intercostal space to access the aorta. A non-absorbable 7–0 silk suture was tied around the aorta between the right innominate and left carotid arteries. This caused a constriction of approximately 65 % using a 27 gauge-needle as a guide. For sham operations to serve as controls, mice received the same surgical procedure, except that the suture around the aorta was removed prior to closing the chest cavity. At various time points (1, 2 and 4 weeks) mice were sacrificed with an overdose of pentobarbital (150 mg/kg). The hearts and lungs were dissected to measure organ weight and to perform histological analysis.

### Type 1 DM model

Ten-week-old male C57BL/6 mice were purchased and injected intraperitoneally with streptozocin (STZ) (Sigma Chemicals, St. Louis, USA) 70 mg/kg/day for 5 days. STZ was dissolved in 10 mmol/L sodium citrate buffer (pH 4.5). Control mice were injected with the buffer alone. Three days later, mice with a random blood glucose level >20 mmol/L were assigned to the hyperglycemic groups, whereas citrate buffer-treated mice were assigned to the control group. Six weeks later, mice were sacrificed with an overdose of pentobarbital (150 mg/kg), and the hearts were harvested for histological and molecular examinations.

### Invasive LV hemodynamic measurements

Thirty minutes or 2 weeks after surgery, mice were anesthetized, intubated, and ventilated as mentioned above. A 1.4F Millar catheter (Millar Instruments, Inc., Houston, TX) was then inserted into the right carotid artery and advanced into the LV cavity. LV systolic pressure (LVSP) and LV end-diastolic pressure (LVEDP), maximum and minimum rates of change in LV pressure (dp/dt max and dp/dt min, respectively) were recorded. The exponential time constant of LV relaxation (τ) was calculated using Power Lab software (blood pressure module; AD Instruments, Shanghai Trading Co, Shanghai, China). Some mice were treated with high glucose or mannitol (2 g/kg ip) and the time-course of LV hemodynamics was recorded. For mice that received a high glucose load test, overnight fasting was performed before the test to avoid xylazine/ketamine anesthesia-induced hyperglycemia [22].

### Blood glucose measurements

Plasma glucose (fasting or in response to high glucose or insulin treatment) measurements were performed in all mice using a standard glucometer (Accu-Chek, Roche, Mannheim, Germany). Whole blood samples (3 μl) were taken from mouse tails with a glucose sensor inserted in the glucometer. Plasma glucose concentrations were read 30 s later. Serum insulin levels were measured according to the protocol provided by the manufacturer (EIA-3440 enzyme linked immunosorbent assay kit; Diagnosis-related Group, Germany). Homeostasis model assessment for insulin resistance (HOMA-IR) values were determined from results of the fasting blood glucose (FBG) and fasting insulin (FINS) tests, using the equation HOMA-IR = [FBG (mg/dl, 1 mmol/L = 18 mg/dL) × FINS (ng/ml)]/22.5.

### Histology examination

The extent of myocardial fibrosis was measured, as described elsewhere [21, 23]. Hearts were fixed with 10 % formalin, and each heart was cut into four sections and stained with Masson trichrome.

### Cell culture

Ventricular myocytes were prepared from Sprague–Dawley rats (age, 1–2 days), which were obtained from the Animal Center of Southern Medical University. In brief, the rats were sacrificed by 2 % isoflurane inhalation. The hearts were quickly excised and immediately embedded in freezing Hank’s solution. Cardiomyocytes were dispersed by digestion with 0.1 % trypsin and 0.03 % collagenase at 37 °C. The cells were then collected after differential adhesion of non-cardiomyocytes and plated at a density of 150–200 cells/mm^2^. Cardiomyocytes were incubated for 48 h in Dulbecco’s modified Eagle’s medium supplemented with 10 % fetal calf serum and then grown for 24 h under serum free conditions. Cells were treated with angiotensin II (1 μmol/L) and/or high-glucose solution (25/50 mmol/L), and collected for protein extraction at 24 h.

### Western blotting

Protein was obtained from cultured cardiomyocytes or hearts from STZ-treated mice, and was extracted in radio-immunoprecipitation assay lysis buffer. Samples were loaded onto 10 % sodium dodecyl sulfate–polyacrylamide gels and the protein was transferred to polyvinyl difluoride membranes. Immunoblotting was then performed by using PGC-1α antibody (ab191838; Abcam, Shanghai, China) or autophagy antibodies (light chain 3 beta [LC3B]-I/II, 2775s; and P62, 5114s; Cell Signaling Technology, Danvers, MA), and GAPDH antibody (ARG10112; Arigo, Taiwan, China). Immunoreactive bands were visualized by the enhanced chemiluminescence method (Amersham, Piscataway, NJ) with a western blotting detection system (Kodak Digital Science, Rochester, NY). These bands were quantified by densitometry with Scion Image software (Image J 1.42q; NIH, Bethesda, MD). We used the LC3B-II/loading control ratio rather than the LC3B II/LC3B-I ratio for qualification of LC3-II expression levels according to a newly published guideline [24].

### Assay of autophagic flux

To measure autophagic flux, cardiomyocytes were treated with bafilomycin A1 (Selleck; Texas, USA), a lysosomal inhibitor, at 100 nmol/L for 24 h. Cellular autophagic flux was estimated by western blots of LC3B-II and P62 protein as well as by tandem fluorescent mRFP-GFP-LC3. Cultured cardiomyocytes were infected with lentivirus carrying mRFP-GFP-LC3 (Cat. No. GPL2001; Genechem Co., Shanghai, China) for 72 h (multiple of infection = 25). subsequently, cells were either treated with angiotensin II (1 μmol/L), glucose (5.5 or 25 μmol/L), or bafilomycin A1 (100 nmol/L) for 24 h. Cells were then washed with PBS, fixed with 4 % paraformaldehyde and viewed under confocal laser scanning microscopy (Olympus FV1000; Japan). In merged images, puncta in autophagosome appeared yellow, while puncta in autolysosomes appeared red.

### Statistical analysis

All data analyses were performed using SPSS 17.0 software (SPSS, Inc., Chicago, IL). Data are presented as mean ± standard error of the mean. Comparisons were made using unpaired Student’s t tests and one-way or two-way ANOVA, as appropriate. P-values less than 0.05 were considered statistically significant.

## Results

### Diastolic dysfunction appears in the early phase of pressure overload

**Fig. 1:**
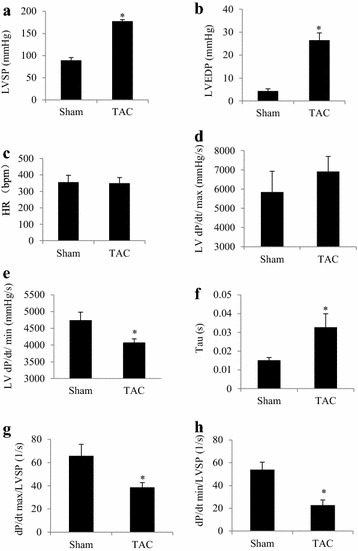
Diastolic dysfunction appears in the early phase of pressure overload. After 30 min of transverse aortic constriction (TAC) in C57 mice, invasive hemodynamics were evaluated. **a** Left ventricular systolic pressure (LVSP). **b** Left ventricular end-diastolic pressure (LVEDP). **c** Heart rate. **d** Maximum rate of change in left ventricular pressure (dp/dt max). **e** Minimum rate of change in left ventricular pressure (dp/dt min). **f** Exponential time constant of relaxation (τ). **g** dp/dt max corrected with LVSP. **h** dp/dt min corrected with LVSP. *P < 0.05 versus the sham group, n = 6 and 9 in the sham and TAC groups, respectively

In mice with TAC for 30 min, invasive evaluation of LV hemodynamics using a Millar catheter. In comparison with sham group, LVEDP and the exponential time constant of LV relaxation (τ) were greater, while LV dp/dt min was smaller in TAC group. These findings indicated damage of diastolic function (Fig. 1a–h).

### Diastolic dysfunction and insulin resistance appear in mice with cardiac hypertrophy

**Fig. 2:**
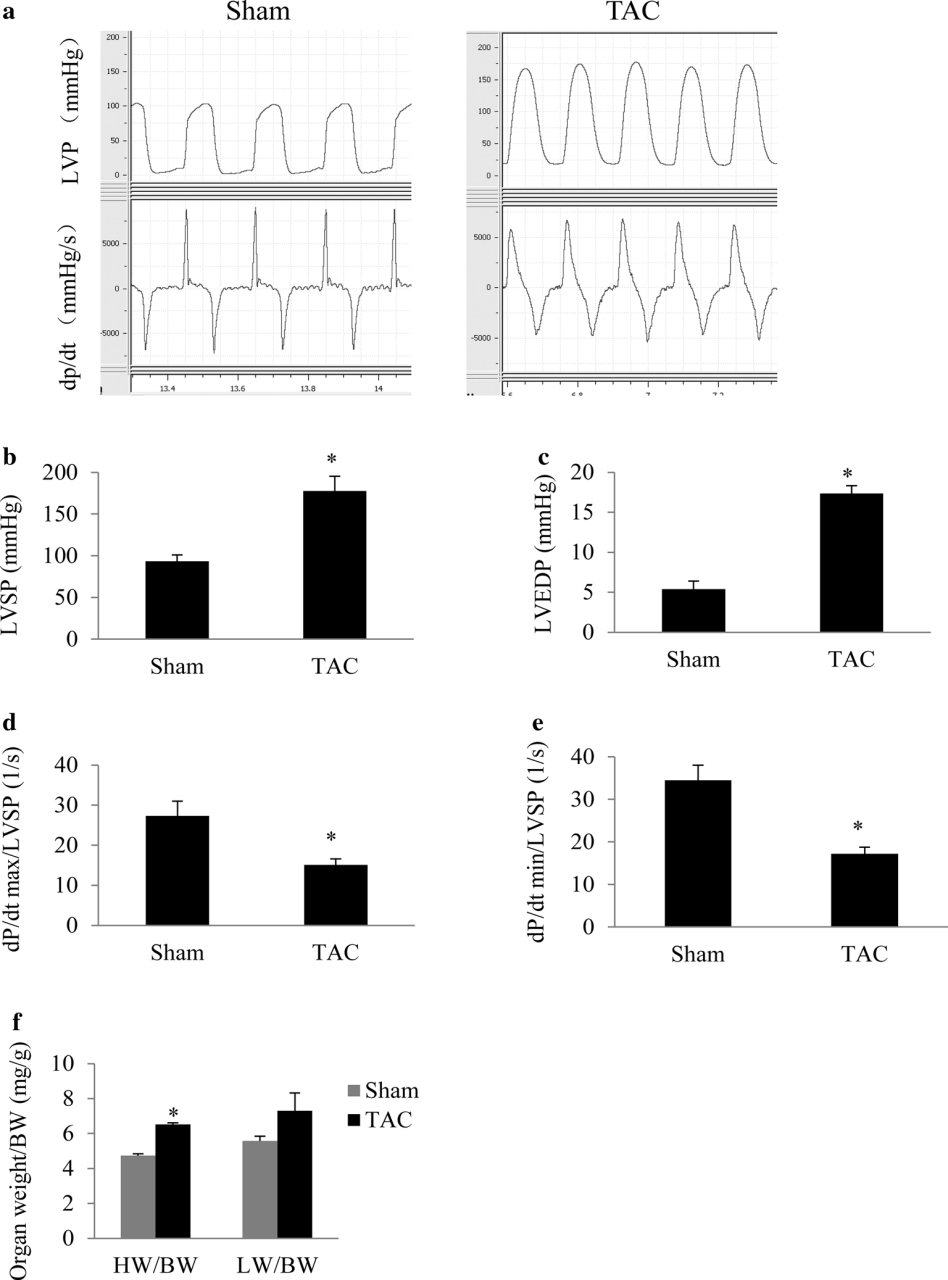
Diastolic dysfunction and insulin resistance appear in mice with cardiac hypertrophy. After 2 weeks of TAC in C57 mice, invasive hemodynamics were evaluated. **a** Representative recordings of left ventricular pressure and the rate of change in pressure. **b** Left ventricular systolic pressure (LVSP). **c** Left ventricular end-diastolic pressure (LVEDP). **d** Maximum rate of change in left ventricular pressure (dp/dt max) corrected by LVSP. **e** Minimum rate of change in left ventricular pressure (dp/dt min) corrected by LVSP. **f** Heart to body weight ratio (HW/BW) and lung to body weight ratio (LW/BW). *P < 0.05 versus the sham group, n = 9 in each group

**Fig. 3:**
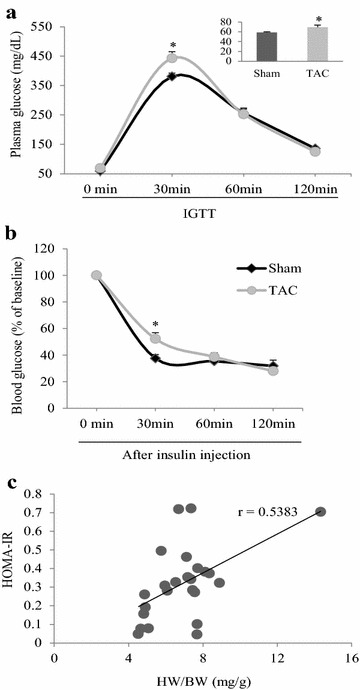
Insulin resistance appears in mice with TAC for 2 weeks. **a** Plasma glucose concentrations in response to an intraperitoneal glucose tolerance test (IGTT, after fasting for 14 h, glucose 2 g/kg, ip). The insert shows fasting glucose levels. **b** Time course of plasma glucose concentrations after insulin injection. **c** Linear correlation between HOMA-IR and heart weight to body weight ratio (HW/BW) in the TAC and sham mice groups. *P < 0.05 versus the corresponding sham group, n = 16 and 12 in the sham and TAC groups, respectively, (*panels* **a**, **b**); n = 24 for *panel* **c**

In mice with TAC for 2 weeks, the diastolic parameters LVEDP and τ were greater, dp/dt min and dp/dt max corrected by LV systolic pressure was significantly smaller than in the sham group. These findings indicated that chronic TAC can induce diastolic and systolic dysfunction (Fig. 2a–e). Cardiac hypertrophy was identified according to a significant greater heart to body weight ratio than in the sham group (Fig. 2f). The results of an intraperitoneal glucose tolerance test showed significantly higher peak glucose levels in the TAC group than in the sham group (Fig. 3a). This finding suggested glucose tolerance in mice with cardiac hypertrophy. An insulin resistance test showed that plasma glucose concentrations at 30 min after insulin injection were higher in the TAC group than in the sham group (Fig. 3b). Additionally, the insulin resistance index HOMA-IR was significantly correlated with the heart to body weight ratio (r = 0.5383, *P* < 0.01, Fig. 3c).

### Acute hyperglycemic stress suppresses diastolic function in mice with preexistent diastolic dysfunction

**Fig. 4:**
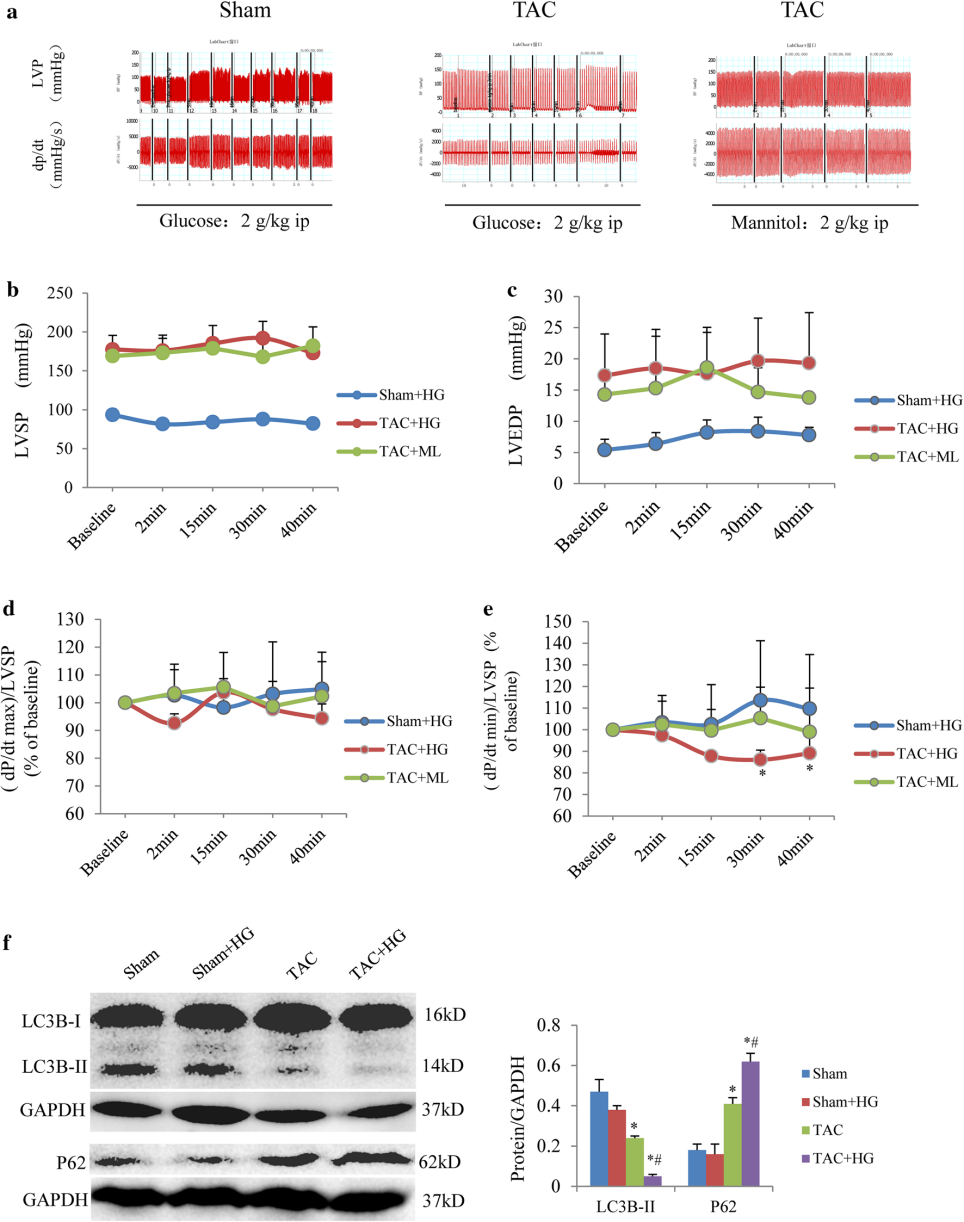
Acute hyperglycemic stress suppresses diastolic function in mice with TAC for 2 weeks. **a** Representative time course recordings of left ventricular pressure (LVP) and the rate of rise and fall rate of LV (dp/dt). **b** Time course of left ventricular systolic pressure (LVSP). **c** Time course of left ventricular end-diastolic pressure (LVEDP). **d** Time course of corrected dp/dt max in response to glucose overload (2 g/kg, ip). **e** Time course of corrected dp/dt min in response to glucose overload. *P < 0.05 vs. the corresponding time point in the sham group, n = 5 per group. **f** Western blot analysis of LC3B-II and P62. *P < 0.05 vs. sham group; ^#^ *P* < 0.05 vs. TAC group, n = 5 per group

In mice with TAC for 2 weeks and sham-operated mice, the time course of LV hemodynamics in response to acute hyperglycemia (glucose 2 g/kg, ip) was evaluated (Fig. 4a–e). We found that corrected LV dp/dt min was significantly decreased at 30 and 40 min after glucose overload in TAC mice, but not in sham mice (Fig. 4e). There was no significant change in corrected dp/dt max in the TAC and sham groups (Fig. 4d). Injection of mannitol with the same osmolarity as high glucose in TAC mice had no significant effect on LV hemodynamics (Fig. 4a–e). Autophagic marker proteins were also examined. LC3B-II was lower and P62 was higher in the TAC group than in the sham group. A high glucose load further suppressed LC3B-II and increased P62 in TAC mice (Fig. 4f).

### High glucose levels downregulate PGC-1α and inhibit autophagic flux

**Fig. 5:**
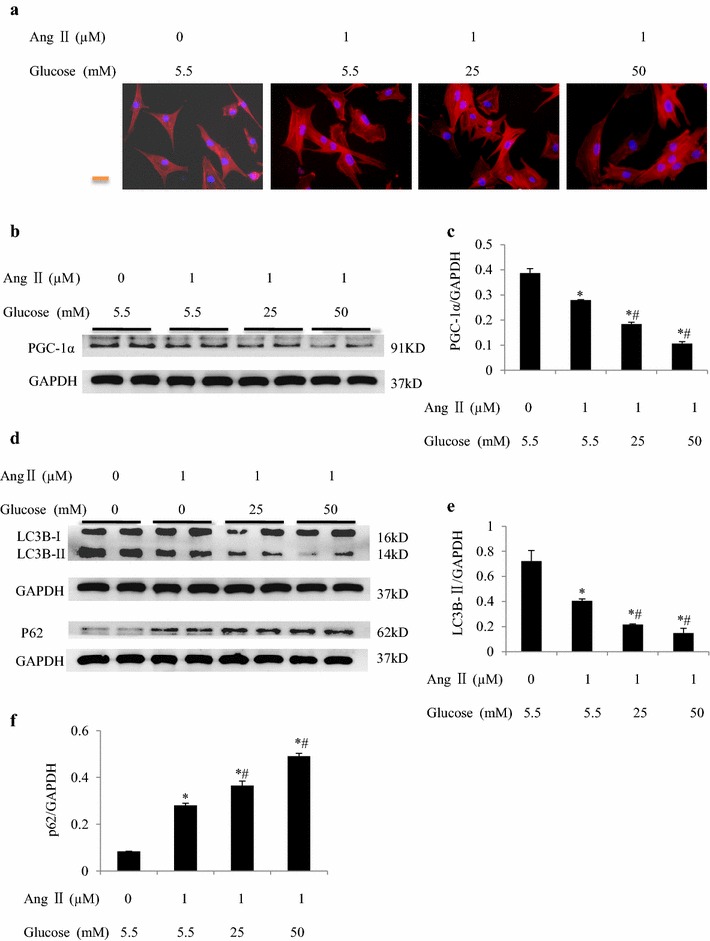
High glucose levels downregulate PGC-1α and inhibit autophagic flux. **a** Representative pictures of cultured neonatal rat cardiomyocytes (NRCs) stained with β-actin plus DAPI staining of the nucleus in response to angiotensin II stimulation with/without high glucose stimulation. Western blot analysis of PGC-1α (**b**, **c**), LC3B-II (**d**, **e**), and P62 (**d**, **f**) expression in cultured NRCs. *P < 0.05 vs. control group, ^#^ *P* < 0.05 vs. angiotensin II group. Each experiment was repeated four times

**Fig. 6:**
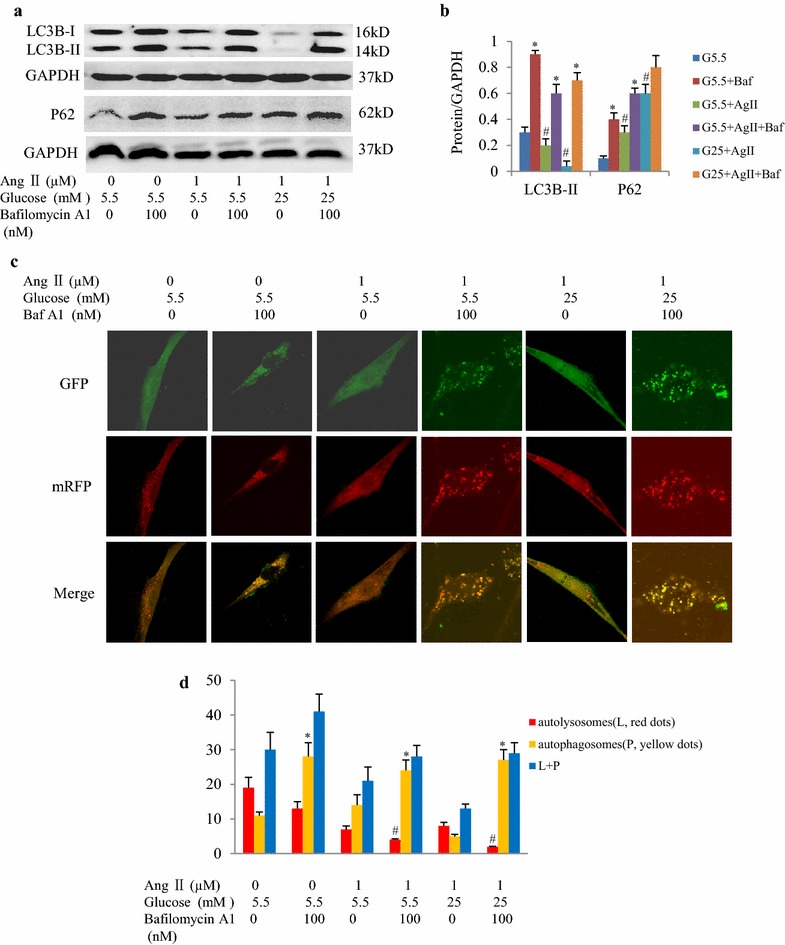
High glucose levels inhibit autophagic flux in cultured neonatal rat cardiomyocytes. **a** Western blot images of LC3B-II and P62 in cultured neonatal rat cardiomyocytes (NRCs) in the presence of bafilomycin A1. **b** Semi-quantitation of LC3B-II and P62 expression levels. **c** Representative immunofluorescent NRCs expressing mRFP-GFP-LC3. **d** Semi-quantitation of autophagosomes (*yellow*) and autolysosomes (*red*). *P < 0.05 vs. the autophagosomes in the corresponding group without bafilomycin A1 treatment; ^#^ *P* < 0.05 vs. the autolysosomes in the corresponding group without bafilomycin A1 treatment. Experiments were repeated four times. G5.5, glucose 5.5 mmol/L; G25, glucose 25 mmol/L; Ang II, angiotensin II; Baf, bafilomycin A1

We then investigated potential mechanisms related to autophagy in cultured neonatal rat cardiomyocytes. In cardiomyocytes, high glucose levels (≥25 mmol/L) enhanced angiotensin II-stimulated cell growth (Fig. 5a), significantly downregulated PGC-1α and LC3B-II, and upregulated the ubiquitin-binding autophagy receptor P62 (Fig. 5b–f). An autophagic flux assay showed that bafilomycin A1 increased LC3B-II and P62 protein levels in the presence of angiotensin II and high glucose levels (Fig. 6a, b). A tandem fluorescent mRFP-GFP-LC3 assay showed that angiotensin II and high glucose levels increased the ratio of autophagosomes to autolysosomes, which was enhanced by cotreatment with bafilomycin A1 (Fig. 6c, d). These results suggested that high glucose levels damaged mitochondrial energy metabolism and inhibited autophagic flux in the presence of the prohypertrophic factor angiotensin II.

### Chronic hyperglycemia induces LV diastolic dysfunction and inhibits autophagic flux

**Fig. 7:**
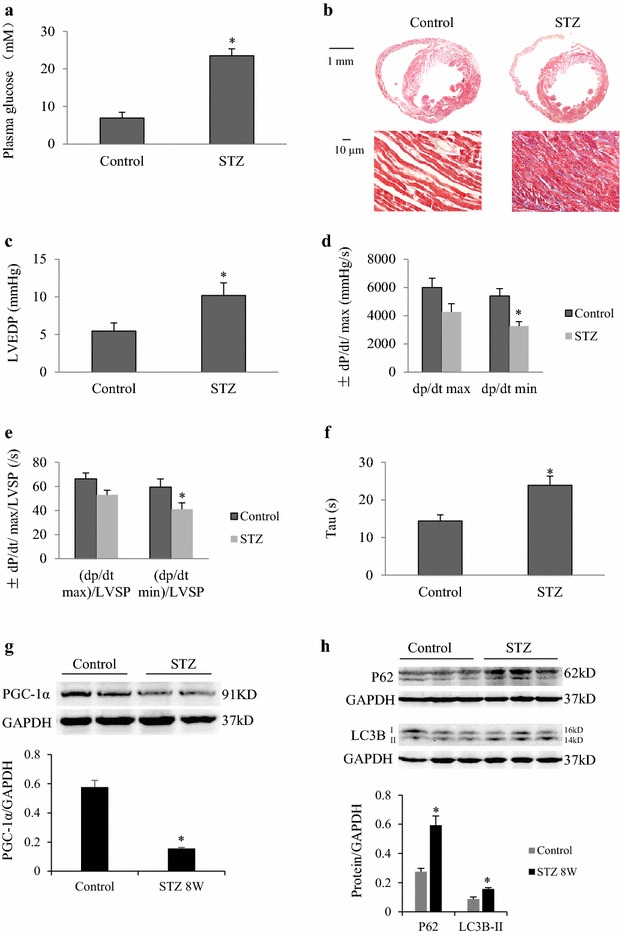
Chronic hyperglycemia levels induce LV diastolic dysfunction and inhibit autophagic flux in mice at 6 weeks after induction of type 1 DM. **a** Plasma glucose concentrations. **b** Representative images of H&E (*top*) and Masson stained (*bottom*) myocardial tissue. Scale bar = 10 µm. **c** Left ventricular end-diastolic pressure (LVEDP). **d** Maximum of change in left ventricular pressure (dp/dt max). **e** Minimum rate of change in left ventricular pressure (dp/dt min). **f** Exponential time constant of relaxation (τ). **g, h** Western blot analysis of PGC-1α, LC3B-II, and P62 expression in myocardial tissues. *P < 0.05 vs. the sham group, n = 5 per group

In mice with STZ-induced chronic hyperglycemia, plasma glucose levels, myocardial fibrosis, diastolic function and autophagic flux were evaluated 6 weeks later. In STZ-treated mice, mean blood glucose levels reached as high as 23 mmol/L (Fig. 7a). Additionally, myocardial fibrosis appeared in the heart (Fig. 7b), and LV diastolic dysfunction was detected as shown by an increase in LVEDP and τ as well as a decrease in dp/dt min (Fig. 7c–f). Similar to the findings in high glucose-treated cardiomyocytes, we found that PGC-1α was downregulated, while LC3B-II and P62 were upregulated in the heart of STZ-treated mice (Fig. 7g).

## Discussion

**Fig. 8:**
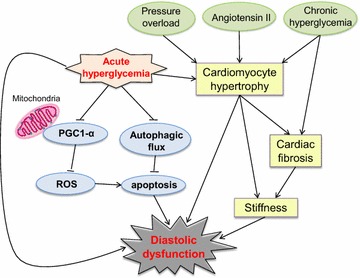
Proposed hyperglycemia-mediated diastolic dysfunction signaling pathway. The *solid* and *dotted arrows* indicate evidence from our study and that from previous literature, respectively. *ROS* reactive oxygen species; *uparrow* activation and;  inhibition

LV diastolic dysfunction can be induced in patients with hypertension, myocardial hypertrophy, or DM. These disorders may also co-exist in patients with metabolism syndrome, which is likely to accelerate diastolic dysfunction. Our previous studies demonstrated that better glucose control with voglibose, an alpha-glycosidase inhibitor, or vildagliptin, a dipeptidyl-peptidase IV inhibitor, improves heart failure in mice with TAC [3, 25]. In patients with acute myocardial infarction, acute hyperglycemic stress at admission predicts poor long-term prognosis [12, 26], but the mechanisms are not clearly understood. Diastolic dysfunction is a critical prognostic factor for cardiovascular and some non-cardiovascular diseases [5, 9]. However, the effect of acute hyperglycemia on the hypertrophied heart with preexistent diastolic dysfunction and the mechanisms involved remain unclear. In this study, we found that acute hyperglycemia suppressed diastolic function in the hypertrophied heart, downregulated PGC-1α, a master regulator of mitochondrial biogenesis and function, and inhibited autophagic flux (see a summary in Fig. 8).

Acute hyperglycemia is a transient hyperglycemia referred to as stress-induced hyperglycemia. Acute hyperglycemia is commonly observed on admission and during hospitalization for cardiovascular diseases, and non-cardiovascular diseases, such as traumatic injury, burns and surgical intervention. This condition is associated with an increase in morbidity and mortality compared with hospitalized patients with normal glucose levels [27]. A recent report by Baranyai et al. [28] demonstrated that cardioprotection exerted by remote ischemic preconditioning can be abolished by acute hyperglycemia. Mebazaa et al. [29] reported that hyperglycemia is a prognostic predictor for mortality of patients with acute heart failure. They found a 9 % increase in the risk of 30-day mortality for every 1 mmol/L increase in blood glucose concentration in patients with acute heart failure.

With regard to the effect of chronic hyperglycemia on the heart, Rubin et al. [30] reported that chronic hyperglycemia contributes to subclinical myocardial damage in persons without clinically evident coronary heart disease. This clinical finding is in agreement with our results that STZ-induced chronic hyperglycemia suppressed LV diastolic function in mice without preexistent cardiovascular disease.

Pre-clinical diastolic dysfunction is prevalent and will progress to symptomatic heart failure [31]. In patients with DM or hypertension, diastolic dysfunction is a risk of progression to heart failure and death [32, 33]. In agreement with previous studies [3, 34], in our study, we found that fasting glucose and glucose tolerance were impaired in hypertensive mice induced by TAC. This situation may be one of the reasons for diastolic dysfunction. Catena et al. [35] reported that impaired fasting glucose and glucose tolerance were associated with more prominent diastolic impairment in uncomplicated hypertensive patients, which is in consist with findings in our study.

Mitochondrial dysfunction of cardiomyocytes has been implicated in heart failure of diverse etiologies. PGC-1α is a master regulator of mitochondrial biogenesis and breathing, and downregulation or loss of PGC-1α is detrimental for heart failure [36, 37]. Hyperglycemia-induced reactive oxygen species in mitochondria play a critical role in the development of complications from diabetes. Overexpression of PGC-1α completely blocks hyperglycemia-induced production of mitochondrial reactive oxygen species and promotion of mitochondrial biogenesis [38]. Based on these lines of evidence, our study suggests high glucose-downregulated PGC-1α contributes to diastolic dysfunction. Recent studies have shown that stimulating mitochondrial function or control of glucose with voglibose or sitagliptin improves diastolic dysfunction [39, 40].

Diastolic dysfunction exists in the hypertrophied heart. Autophagy is diminished in response to pressure overload or β-adrenergic stimulation, although protein turnover is increased during hypertrophy [41, 42]. Wang et al. [43] reported that low concentrations of angiotensin II induce autophagy while high concentrations diminish autophagy in cultured cardiomyocytes, which is in agreement with our results. Appropriated levels of autophagic flux are thought to maintain cellular homeostasis and cell survival, which are beneficial for heart failure [44, 45]. Numerous studies have demonstrated that autophagy is impaired in the heart under DM [45], which is also in agreement with our findings. Zhang et al. [46] recently reported that an increase in autophagy prevents cardiac fibrosis and inflammation in type 1 DM mice. Another study showed that caloric restriction improves diastolic dysfunction in diabetic rat hearts by enhancing autophagy [47]. In our study, we observed that acute stimulation with high glucose levels rapidly downregulated LC3B-II and upregulated P62. This finding suggested inhibition of an autophagy by reducing formation of autophagosomes. Zhang et al. [48] reported that induction of autophagosome can be as fast as 15 min after treatment of retinal pigment epithelial cells with the drug N-retinyl-N-retinylidene ethanolamine.

In contrast to acute hyperglycemia, chronic hyperglycemia increased myocardial LC3B-II level. This situation could be due to long-term inhibition of autophagic flux as shown by upregulation of P62. Kanamori et al. [49] reported diastolic impairment and in increase of LC3 II and P62 in mice with type 1 DM, which is consistent with our findings. However, the differences between acute and chronic hyperglycemia on autophagic activity need to be further clarified. Taken together, our findings suggest that reactivation of autophagy is likely to improve diastolic dysfunction induced by myocardial hypertrophy and DM.

## Conclusion

Acute hyperglycemia suppresses diastolic function, damages mitochondrial energy signaling, and inhibits autophagic flux in prohypertrophic factor-stimulated cardiomyocytes. Our findings suggest that preventing acute hyperglycemia is of clinical importance for avoiding deterioration of diastolic dysfunction in patients with LV hypertrophy. Modulation of autophagy may be a novel strategy for improving diastolic dysfunction induced by myocardial hypertrophy and DM.

## Abbreviations

LV: left ventricular
TAC: transverse aortic constriction
PGC)-1α: peroxisome proliferator-activated receptor-γ coactivator 1α
DM: diabetes mellitus
LC3B-II: light chain 3 beta-II
P62: ubiquitin-binding autophagy receptor
STZ: streptozocin
LVSP: LV systolic pressure
LVEDP: LV end diastolic pressure
HOMA-IR: homeostasis model assessment for insulin resistance
